# 4,4′-Bis(2,2-diphenyl­vin­yl)-1,1′-biphen­yl

**DOI:** 10.1107/S1600536810052840

**Published:** 2010-12-24

**Authors:** Hong-Ni Liu, Gao Zhang, Lan Hu, Peng-Fei Su, Yun-Feng Li

**Affiliations:** a Xi’an Modern Chemistry Research Institute, Xi’an 710065, People’s Republic of China; b Xi’an Caijing Opto-Electrical Science & Technology Co. Ltd, Xi’an 710065, People’s Republic of China

## Abstract

The title mol­ecule, C_40_H_30_, lies on an inversion center. The two unique phenyl rings form dihedral angles of 51.98 (8) and 67.58 (8)° with the essentially planar biphenyl unit [maximum deviation = 0.0360 (14) Å].

## Related literature

For applications of the title compound, see: Park *et al.* (2005 ▶); Kim *et al.* (2009 ▶). For the preparation of the title compound, see: Zheng *et al.* (2004 ▶).
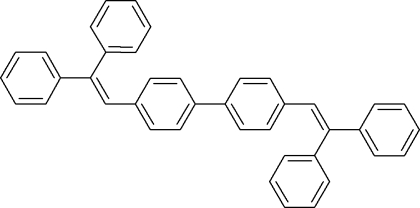


## Experimental

### 

#### Crystal data

C_40_H_30_
*M* *_r_* = 510.7
Monoclinic, 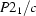

*a* = 9.277 (2) Å
*b* = 14.625 (3) Å
*c* = 10.460 (2) Å
β = 92.669 (4)°
*V* = 1417.6 (5) Å^3^
*Z* = 2
Mo *K*α radiation
μ = 0.07 mm^−1^
*T* = 296 K
0.39 × 0.25 × 0.18 mm

#### Data collection

Bruker SMART CCD diffractometer
6984 measured reflections
2508 independent reflections
1479 reflections with *I* > 2σ(*I*)
*R*_int_ = 0.030

#### Refinement

*R*[*F* ^2^ > 2σ(*F* ^2^)] = 0.043
*wR*(*F* ^2^) = 0.110
*S* = 0.94
2508 reflections
181 parameters
H-atom parameters constrained
Δρ_max_ = 0.12 e Å^−3^
Δρ_min_ = −0.18 e Å^−3^

### 

Data collection: *SMART* (Bruker, 1997 ▶); cell refinement: *SAINT* (Bruker, 1997 ▶); data reduction: *SAINT*; program(s) used to solve structure: *SHELXS97* (Sheldrick, 2008 ▶); program(s) used to refine structure: *SHELXL97* (Sheldrick, 2008 ▶); molecular graphics: *SHELXTL* (Sheldrick, 2008 ▶); software used to prepare material for publication: *SHELXTL*.

## Supplementary Material

Crystal structure: contains datablocks I, global. DOI: 10.1107/S1600536810052840/lh5185sup1.cif

Structure factors: contains datablocks I. DOI: 10.1107/S1600536810052840/lh5185Isup2.hkl

Additional supplementary materials:  crystallographic information; 3D view; checkCIF report

## Figures and Tables

**Fig. 1.**
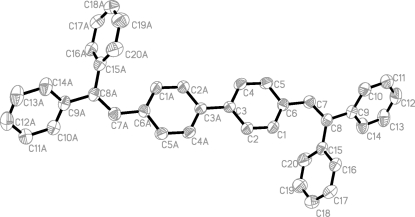
The molecular structure of the title compound, with atom labels and 30% probability displacement ellipsoids for non-H atoms (symmetry code (A): 1-x, 2-y, -z).

## supplementary crystallographic information

### Comment

Distyrylarylene (DSA) derivatives have been widely investigated because of there high thermal stability and good film forming ability. The title compound has been used to fabricate white organic light-emitting diodes (WOLEDs)(Kim *et al.*, 2009; Park *et al.*, 2005). The synthesis and luminescent properties of DPVBi have already been described (Zheng, *et al.* 2004). The molecular structure of the title compound is shown in Fig.1. The molecule lies on an inversion center. The two unique phenyl rings form dihedral angles of 51.98 (8) [for C9-C14] and 67.58 (8)° [for C15-C20] with the essentially planar biphenyl unit [maximum deviation = 0.0360 (14)Å]

### Experimental

The synthesis of the crude product was carried out according to reported methods (Zheng, *et al.* 2004). Suitable crystals were obtained by evaporation of a tetrahydrofuran/methanol (1:9, *v*/*v*) solution of the title compound at room temperature. Spectroscopic analysis: IR(KBr, cm^-1^): 3020,1597,1494,1441,762,697,815; ^1^H NMR (CDCl_3_, δ, p.p.m.): 7.3 (s, 20H), 6.9—7.2 (m, 10 H).

### Refinement

All H atoms were positioned geometrically and refined as riding [C—H = 0.93Å; U_iso_(H) = 1.2U_eq_(C)].

### Crystal data

**Table:**

C_40_H_30_	*F*(000) = 540
*M**_r_* = 510.7	*D*_x_ = 1.196 Mg m^−^^3^
Monoclinic, *P*2_1_/*c*	Melting point: 477 K
Hall symbol: -P 2ybc	Mo *K*α radiation, λ = 0.71073 Å
*a* = 9.277 (2) Å	Cell parameters from 1536 reflections
*b* = 14.625 (3) Å	θ = 2.4–25.1°
*c* = 10.460 (2) Å	µ = 0.07 mm^−^^1^
β = 92.669 (4)°	*T* = 296 K
*V* = 1417.6 (5) Å^3^	Block, yellow
*Z* = 2	0.39 × 0.25 × 0.18 mm

### Data collection

**Table:**

Bruker SMART CCD diffractometer	1479 reflections with *I* > 2σ(*I*)
Radiation source: fine-focus sealed tube	*R*_int_ = 0.030
graphite	θ_max_ = 25.1°, θ_min_ = 2.4°
φ and ω scans	*h* = −11→10
6984 measured reflections	*k* = −17→17
2508 independent reflections	*l* = −12→10

### Refinement

**Table:**

Refinement on *F*^2^	Primary atom site location: structure-invariant direct methods
Least-squares matrix: full	Secondary atom site location: difference Fourier map
*R*[*F*^2^ > 2σ(*F*^2^)] = 0.043	Hydrogen site location: inferred from neighbouring sites
*wR*(*F*^2^) = 0.110	H-atom parameters constrained
*S* = 0.94	*w* = 1/[σ^2^(*F*_o_^2^) + (0.0637*P*)^2^] where *P* = (*F*_o_^2^ + 2*F*_c_^2^)/3
2508 reflections	(Δ/σ)_max_ < 0.001
181 parameters	Δρ_max_ = 0.12 e Å^−^^3^
0 restraints	Δρ_min_ = −0.18 e Å^−^^3^

### Special details

**Table:**

Geometry. All e.s.d.'s (except the e.s.d. in the dihedral angle between two l.s. planes) are estimated using the full covariance matrix. The cell e.s.d.'s are taken into account individually in the estimation of e.s.d.'s in distances, angles and torsion angles; correlations between e.s.d.'s in cell parameters are only used when they are defined by crystal symmetry. An approximate (isotropic) treatment of cell e.s.d.'s is used for estimating e.s.d.'s involving l.s. planes.
Refinement. Refinement of *F*^2^ against ALL reflections. The weighted *R*-factor *wR* and goodness of fit *S* are based on *F*^2^, conventional *R*-factors *R* are based on *F*, with *F* set to zero for negative *F*^2^. The threshold expression of *F*^2^ > σ(*F*^2^) is used only for calculating *R*-factors(gt) *etc*. and is not relevant to the choice of reflections for refinement. *R*-factors based on *F*^2^ are statistically about twice as large as those based on *F*, and *R*- factors based on ALL data will be even larger.

### Fractional atomic coordinates and isotropic or equivalent isotropic displacement parameters (Å^2^)

**Table:**

	*x*	*y*	*z*	*U*_iso_*/*U*_eq_	
C1	0.69791 (18)	0.84611 (10)	0.10768 (16)	0.0652 (5)	
H1	0.6951	0.7855	0.1343	0.078*	
C2	0.57204 (17)	0.88788 (10)	0.06689 (14)	0.0625 (4)	
H2	0.4864	0.8549	0.0677	0.075*	
C3	0.56784 (15)	0.97810 (9)	0.02422 (13)	0.0488 (4)	
C4	0.69840 (17)	1.02440 (10)	0.03150 (16)	0.0633 (5)	
H4	0.7009	1.0854	0.0066	0.076*	
C5	0.82414 (17)	0.98268 (10)	0.07443 (16)	0.0642 (5)	
H5	0.9088	1.0168	0.0795	0.077*	
C6	0.82915 (16)	0.89105 (10)	0.11058 (13)	0.0539 (4)	
C7	0.96931 (16)	0.85159 (11)	0.15032 (14)	0.0609 (4)	
H7	1.0411	0.8943	0.1703	0.073*	
C8	1.01194 (17)	0.76349 (10)	0.16283 (14)	0.0570 (4)	
C9	1.16235 (17)	0.74195 (11)	0.20673 (15)	0.0599 (4)	
C10	1.2386 (2)	0.79640 (12)	0.29442 (18)	0.0746 (5)	
H10	1.1933	0.8464	0.3300	0.090*	
C11	1.3807 (2)	0.77781 (13)	0.33001 (19)	0.0849 (6)	
H11	1.4301	0.8155	0.3887	0.102*	
C12	1.4498 (2)	0.70404 (14)	0.27931 (19)	0.0835 (6)	
H12	1.5463	0.6923	0.3014	0.100*	
C13	1.3742 (2)	0.64829 (16)	0.19597 (19)	0.0928 (6)	
H13	1.4194	0.5974	0.1625	0.111*	
C14	1.2324 (2)	0.66610 (13)	0.16054 (17)	0.0809 (5)	
H14	1.1828	0.6265	0.1046	0.097*	
C15	0.91607 (18)	0.68499 (10)	0.12923 (15)	0.0588 (4)	
C16	0.86251 (18)	0.62995 (10)	0.22408 (15)	0.0630 (4)	
H16	0.8899	0.6411	0.3093	0.076*	
C17	0.7692 (2)	0.55891 (11)	0.19388 (18)	0.0730 (5)	
H17	0.7328	0.5235	0.2588	0.088*	
C18	0.7301 (2)	0.54030 (12)	0.0686 (2)	0.0828 (6)	
H18	0.6666	0.4927	0.0485	0.099*	
C19	0.7846 (2)	0.59197 (14)	−0.02639 (18)	0.0935 (6)	
H19	0.7599	0.5786	−0.1115	0.112*	
C20	0.8763 (2)	0.66401 (12)	0.00322 (17)	0.0815 (6)	
H20	0.9120	0.6991	−0.0624	0.098*	

### Atomic displacement parameters (Å^2^)

**Table:**

	*U*^11^	*U*^22^	*U*^33^	*U*^12^	*U*^13^	*U*^23^
C1	0.0611 (11)	0.0537 (9)	0.0803 (12)	0.0013 (9)	−0.0031 (9)	0.0190 (8)
C2	0.0585 (11)	0.0566 (10)	0.0719 (11)	−0.0035 (8)	−0.0022 (8)	0.0120 (8)
C3	0.0532 (9)	0.0464 (8)	0.0472 (8)	0.0022 (7)	0.0066 (7)	−0.0038 (6)
C4	0.0540 (11)	0.0446 (8)	0.0918 (12)	0.0042 (8)	0.0095 (9)	0.0033 (8)
C5	0.0487 (10)	0.0507 (9)	0.0937 (12)	0.0002 (8)	0.0088 (9)	−0.0001 (8)
C6	0.0510 (10)	0.0550 (9)	0.0558 (9)	0.0048 (8)	0.0044 (7)	0.0019 (7)
C7	0.0555 (11)	0.0601 (10)	0.0673 (10)	0.0027 (8)	0.0046 (8)	0.0031 (8)
C8	0.0604 (10)	0.0573 (10)	0.0536 (10)	0.0061 (8)	0.0068 (7)	0.0043 (7)
C9	0.0627 (11)	0.0613 (10)	0.0560 (9)	0.0105 (8)	0.0052 (8)	0.0096 (8)
C10	0.0694 (13)	0.0656 (11)	0.0882 (13)	0.0097 (9)	−0.0017 (10)	0.0013 (9)
C11	0.0693 (13)	0.0806 (13)	0.1033 (15)	0.0018 (11)	−0.0101 (11)	0.0094 (11)
C12	0.0641 (12)	0.1014 (15)	0.0855 (15)	0.0166 (12)	0.0084 (11)	0.0284 (12)
C13	0.0890 (16)	0.1085 (16)	0.0810 (14)	0.0430 (13)	0.0047 (12)	0.0004 (12)
C14	0.0833 (14)	0.0875 (13)	0.0713 (12)	0.0301 (11)	−0.0033 (10)	−0.0057 (10)
C15	0.0696 (11)	0.0540 (9)	0.0531 (10)	0.0130 (8)	0.0038 (8)	0.0019 (7)
C16	0.0745 (11)	0.0573 (9)	0.0572 (10)	0.0107 (9)	0.0035 (8)	0.0040 (8)
C17	0.0866 (13)	0.0547 (10)	0.0784 (13)	0.0044 (9)	0.0127 (10)	0.0072 (9)
C18	0.0938 (15)	0.0632 (11)	0.0905 (15)	−0.0015 (10)	−0.0057 (12)	−0.0074 (11)
C19	0.1305 (18)	0.0808 (13)	0.0676 (12)	−0.0064 (13)	−0.0119 (12)	−0.0094 (11)
C20	0.1173 (17)	0.0712 (12)	0.0558 (11)	−0.0056 (11)	0.0041 (10)	0.0024 (9)

### Geometric parameters (Å, °)

**Table:**

C1—C2	1.368 (2)	C10—H10	0.9300
C1—C6	1.383 (2)	C11—C12	1.374 (2)
C1—H1	0.9300	C11—H11	0.9300
C2—C3	1.3929 (19)	C12—C13	1.363 (3)
C2—H2	0.9300	C12—H12	0.9300
C3—C4	1.387 (2)	C13—C14	1.375 (3)
C3—C3^i^	1.481 (3)	C13—H13	0.9300
C4—C5	1.373 (2)	C14—H14	0.9300
C4—H4	0.9300	C15—C20	1.386 (2)
C5—C6	1.393 (2)	C15—C16	1.388 (2)
C5—H5	0.9300	C16—C17	1.379 (2)
C6—C7	1.465 (2)	C16—H16	0.9300
C7—C8	1.352 (2)	C17—C18	1.371 (2)
C7—H7	0.9300	C17—H17	0.9300
C8—C9	1.482 (2)	C18—C19	1.364 (3)
C8—C15	1.484 (2)	C18—H18	0.9300
C9—C10	1.384 (2)	C19—C20	1.380 (3)
C9—C14	1.384 (2)	C19—H19	0.9300
C10—C11	1.380 (3)	C20—H20	0.9300
			
C2—C1—C6	122.16 (14)	C12—C11—C10	120.49 (19)
C2—C1—H1	118.9	C12—C11—H11	119.8
C6—C1—H1	118.9	C10—C11—H11	119.8
C1—C2—C3	122.25 (15)	C13—C12—C11	118.78 (18)
C1—C2—H2	118.9	C13—C12—H12	120.6
C3—C2—H2	118.9	C11—C12—H12	120.6
C4—C3—C2	115.70 (14)	C12—C13—C14	121.05 (19)
C4—C3—C3^i^	122.27 (16)	C12—C13—H13	119.5
C2—C3—C3^i^	122.02 (17)	C14—C13—H13	119.5
C5—C4—C3	121.84 (14)	C13—C14—C9	121.11 (19)
C5—C4—H4	119.1	C13—C14—H14	119.4
C3—C4—H4	119.1	C9—C14—H14	119.4
C4—C5—C6	122.23 (15)	C20—C15—C16	117.58 (16)
C4—C5—H5	118.9	C20—C15—C8	121.76 (14)
C6—C5—H5	118.9	C16—C15—C8	120.66 (14)
C1—C6—C5	115.68 (14)	C17—C16—C15	120.99 (16)
C1—C6—C7	126.01 (14)	C17—C16—H16	119.5
C5—C6—C7	118.30 (14)	C15—C16—H16	119.5
C8—C7—C6	130.88 (15)	C18—C17—C16	120.27 (16)
C8—C7—H7	114.6	C18—C17—H17	119.9
C6—C7—H7	114.6	C16—C17—H17	119.9
C7—C8—C9	119.95 (15)	C19—C18—C17	119.70 (18)
C7—C8—C15	123.00 (14)	C19—C18—H18	120.1
C9—C8—C15	117.00 (13)	C17—C18—H18	120.1
C10—C9—C14	117.29 (16)	C18—C19—C20	120.32 (18)
C10—C9—C8	121.86 (14)	C18—C19—H19	119.8
C14—C9—C8	120.85 (16)	C20—C19—H19	119.8
C11—C10—C9	121.19 (17)	C19—C20—C15	121.09 (17)
C11—C10—H10	119.4	C19—C20—H20	119.5
C9—C10—H10	119.4	C15—C20—H20	119.5
			
C6—C1—C2—C3	0.7 (2)	C8—C9—C10—C11	−177.03 (15)
C1—C2—C3—C4	−3.0 (2)	C9—C10—C11—C12	−0.4 (3)
C1—C2—C3—C3^i^	177.73 (16)	C10—C11—C12—C13	−1.7 (3)
C2—C3—C4—C5	1.9 (2)	C11—C12—C13—C14	1.4 (3)
C3^i^—C3—C4—C5	−178.81 (15)	C12—C13—C14—C9	1.0 (3)
C3—C4—C5—C6	1.4 (2)	C10—C9—C14—C13	−3.0 (3)
C2—C1—C6—C5	2.6 (2)	C8—C9—C14—C13	176.71 (15)
C2—C1—C6—C7	−178.80 (14)	C7—C8—C15—C20	71.1 (2)
C4—C5—C6—C1	−3.7 (2)	C9—C8—C15—C20	−106.38 (18)
C4—C5—C6—C7	177.62 (14)	C7—C8—C15—C16	−108.71 (17)
C1—C6—C7—C8	17.8 (2)	C9—C8—C15—C16	73.86 (18)
C5—C6—C7—C8	−163.63 (15)	C20—C15—C16—C17	−2.1 (2)
C6—C7—C8—C9	−179.39 (14)	C8—C15—C16—C17	177.65 (14)
C6—C7—C8—C15	3.3 (2)	C15—C16—C17—C18	1.3 (2)
C7—C8—C9—C10	34.1 (2)	C16—C17—C18—C19	0.5 (3)
C15—C8—C9—C10	−148.38 (15)	C17—C18—C19—C20	−1.5 (3)
C7—C8—C9—C14	−145.60 (15)	C18—C19—C20—C15	0.6 (3)
C15—C8—C9—C14	31.9 (2)	C16—C15—C20—C19	1.2 (3)
C14—C9—C10—C11	2.7 (2)	C8—C15—C20—C19	−178.61 (16)

Symmetry codes: (i) −*x*+1, −*y*+2, −*z*.
